# DPAGT1-CDG: Functional analysis of disease-causing pathogenic mutations and role of endoplasmic reticulum stress

**DOI:** 10.1371/journal.pone.0179456

**Published:** 2017-06-29

**Authors:** Patricia Yuste-Checa, Ana I. Vega, Cristina Martín-Higueras, Celia Medrano, Alejandra Gámez, Lourdes R. Desviat, Magdalena Ugarte, Celia Pérez-Cerdá, Belén Pérez

**Affiliations:** 1 Centro de Diagnóstico de Enfermedades Moleculares, Centro de Biología Molecular-SO UAM-CSIC, Universidad Autónoma de Madrid, Campus de Cantoblanco, Madrid, Spain; 2 Centro de Investigación Biomédica en Red de Enfermedades Raras (CIBERER), Madrid, Spain; 3 Instituto de Investigación Hospital Universitario La Paz (IdiPAZ), Madrid, Spain; University of Florida, UNITED STATES

## Abstract

Pathogenic mutations in *DPAGT1* are manifested as two possible phenotypes: congenital disorder of glycosylation DPAGT1-CDG (also known as CDG-Ij), and limb-girdle congenital myasthenic syndrome (CMS) with tubular aggregates. UDP-N-acetylglucosamine-dolichyl-phosphate N-acetylglucosamine phosphotransferase (GPT), the protein encoded by *DPAGT1*, is an endoplasmic reticulum (ER)-resident protein involved in an initial step in the N-glycosylation pathway. The aim of the present study was to examine the effect of six variants in *DPAGT1* detected in patients with DPAGT1-CDG, and the role of endoplasmic reticulum stress, as part of the search for therapeutic strategies to use against DPAGT1-CDG. The effect of the six mutations, i.e., c.358C>A (p.Leu120Met), c.791T>G (p.Val264Gly), c.901C>T (p.Arg301Cys), c.902G>A (p.Arg301His), c.1154T>G (p.Leu385Arg), and of the novel mutation c.329T>C (p.Phe110Ser), were examined via the analysis of *DPAGT1* transcriptional profiles and GTP levels in patient-derived fibroblasts. In addition, the transient expression of different mutations was analysed in COS-7 cells. The results obtained, together with those of bioinformatic studies, revealed these mutations to affect the splicing process, the stability of GTP, or the ability of this protein to correctly localise in the ER membrane. The unfolded protein response (UPR; the response to ER stress) was found not to be active in patient-derived fibroblasts, unlike that seen in cells from patients with PMM2-CDG or DPM1-CDG. Even so, the fibroblasts of patients with DPAGT1-CDG seemed to be more sensitive to the stressor tunicamycin. The present work improves our knowledge of DPAGT1-CDG and provides bases for developing tailored splicing and folding therapies.

## Introduction

Protein glycosylation refers to the co- and post-translational covalent attachment of oligosaccharide moieties to newly synthesised proteins. This is a complex, multistep and highly regulated process. Many different proteins comprise the cellular machinery involved, which takes place in the cytosol, endoplasmic reticulum and Golgi apparatus. *DPAGT1* codes for UDP-N-acetylglucosamine-dolichyl-phosphate N-acetylglucosaminephosphotransferase (GPT; EC number 2.7.8.15), an enzyme involved in one of the initial steps of the N-glycosylation pathway. This ER-resident transmembrane protein catalyses the transfer of N-acetylglucosamine from cytosolic UDP-N-acetylglucosamine to dolichol-phosphate, which is also located in the ER membrane. The result is the formation of dolichol-pyrophosphate-N-acetylglucosamine—the carrier of the sugars that are finally attached to proteins in glycosylation. *DPAGT1* defects manifest as two alternative phenotypes: congenital disorder of glycosylation DPAGT1-CDG (MIM: 608093; previously known as CDG-Ij), and limb-girdle congenital myasthenic syndrome (CMS) (MIM 614750) with tubular aggregates. The former is a severe, multisystem disease, with most patients presenting moderate to severe psychomotor disability, microcephaly, hypotonia and epilepsy. The clinical manifestations of CMS, in contrast, include muscle weakness plus minimal or absent craniobulbar symptoms [1,2]. The primary pathogenic mechanism arising through *DPAGT1* mutations that leads to CMS is the hypoglycosylation of acetylcholine receptors in neuron endplates. This is also true for mutations in other genes (e.g., *GFPT1*, *ALG2* and *ALG14*) involved in the N-glycosylation pathway that result in CMS [3].

Glycosylated proteins are involved in many biological processes, including cell signalling, immune defence, and protein folding and stability, etc. [4]. The glycosylation of nascent proteins modifies their physical properties, increasing their stability and directing their folding and quality control in the ER [5,6]. Only properly folded proteins are allowed to reach the Golgi apparatus; incompletely folded proteins are retained in the ER until their folding is complete, otherwise they are translocated back to the cytosol to be degraded by the proteasome system (ERAD, ER-associated protein degradation).

A balance between protein folding and protein degradation needs to be established if ER homeostasis is to be maintained. An excess of improperly folded proteins leads to ER stress and the activation of the unfolded protein response (UPR) [7]. In some CDGs, the accumulation of hypoglycosylated proteins in the ER leads to the moderate, chronic activation of the UPR [8,9,10,11]. Therapies designed to attenuate ER stress and/or enhance the protection offered by the UPR might therefore be useful. Indeed, increasing GRP78 levels in cerebellar neurons to restore ER homeostasis has been proposed as a potential therapeutic target for PMM2-CDG [9]

Improving our knowledge of the pathogenic, molecular mechanisms associated with DPAGT1-CDG may provide information of use in the development of tailored therapeutic strategies. The present work functionally characterises six disease-causing mutations identified in three patients with DPAGT1-CDG, and examines the possible contribution of ER stress in the pathophysiology of this disease.

## Materials and methods

### Cell lines and culture conditions

PMM2-CDG, DPAGT1-CDG, and DPM1-CDG patients included in the study were selected by clinical findings and abnormal serum transferrin pattern analysed by isoelectrofocusing or high-performance liquid. Mutations in PMM2 were sought by Sanger sequencing and patients with non–PMM2-CDG were examined by massive parallel sequencing. DPAGT1-CDG, PMM2-CDG and DPM1-CDG patient-derived fibroblasts (Table 1 and S1 Table) were grown from skin biopsies (taken with informed consent) in minimal essential medium supplemented with 1% glutamine, 10% foetal calf serum, and antibiotics, under standard conditions. GM08680 (Coriell Institute for Medical Research, NIGMS Human Genetic Cell Repository, Camden, New Jersey) and CC2509 (Lonza, Basel, Switzerland) cell were used as healthy controls. All DPAGT1 and DMP1 patients-derived fibroblasts available in the laboratory were selected. In the case of PMM2 derived-fibroblast were included four cases compound heterozygous of a severe pathogenic variant and one destabilizing mutations [12]

**Table 1:** Genotype/phenotype of *DPAGT1* defective patients.

Ref.	Paternal allele	Maternal allele	Phenotype	Reference
P1	c.901C>T (p.Arg301Cys)	c.1154T>G (p.Leu385Arg)	Foetal hypokinesia, facial dysmorphism, hypertrichosis, hypotonia, papilar atrophy, bilateral cochlear impairment	[21]
P2	c.791T>G (p.Val264Gly)	c.358C>A (p.Leu120Met)	Hypotonia (CMS)	[30]
P3	c.902G>A (p.Arg301His)	c.329T>C (p.Phe110Ser)	Hypotonia, muscle weakness, hypoacusia, psychomotor retardation	This work

COS-7 cells were grown in minimal essential medium supplemented with 1% glutamine, 5% foetal calf serum, and antibiotics; these cells were used to overexpress mutant DPAGT1 proteins in transient expression experiments (see below).

### Genetic analysis

Genetic analyses were performed for three patients with DPAGT1-CDG. RNA and/or genomic DNA was extracted from patient-derived fibroblasts or whole blood using the MagNA Pure Compact Kit (Roche Applied Sciences) according to the manufacturer’s instructions. Genetic analysis was performed by conventional Sanger sequencing, or by DNA massive parallel sequencing of either the whole exome or of a customised panel of 43 CDG-associated genes (SureSelect, Agilent, Santa Clara, California). All massive parallel sequencing was performed in a Hiseq2000 sequencer (Illumina, San Diego, California). After alignment of the reads and annotation of single nucleotide variants (SNVs), the latter were filtered by segregation analysis, population frequency and bioinformatic analysis to select pathogenic candidates.

*DPAGT1* transcriptional profile analysis was performed using specific primers for amplifying the entire coding *DPAGT1* cDNA and employing the SuperScript^®^Vilo^™^ cDNA Synthesis Kit (Life Technologies, Carlsbad, California). The primers used for RT-PCR amplification or Sanger sequencing can be provided upon request.

The Ethics Committee of the *Universidad Autónoma de Madrid* approved the present study. Written informed consent from the parents or their guardians was obtained prior to analysis.

### Western blotting

Patient-derived cells were harvested with trypsin and resuspended in lysis buffer (1% triton, 10% glycerol, 150mM NaCl, 10mM trisHCl, pH 7.5) containing Complete Mini EDTA-free Protease Inhibitor Cocktail (Roche Applied Sciences, Mannheim, Germany). Protein amounts were quantified via the Bradford assay (BioRad, Hercules, CA, USA); samples were prepared in NuPage^®^LDS sample buffer 4x (Life Technologies, Carlsbad, California) and dithiothreitol (DTT) and subjected to electrophoresis in 10% NuPAGE Novex Bis-Tris mini gels (Life Technologies, Carlsbad, California). ProSieve Color Protein Markers (Lonza, Basel, Switzerland) were used as molecular weight markers. Proteins were transferred to nitrocellulose membranes using the iBlot Dry Blotting System (Life Technologies, Carlsbad, California). Membranes were blocked for at least 1 h with 0.05% PBS-Tween and 5% low-fat milk. Immunodetection was performed using anti-GPT (Abcam, Cambridge, UK), anti-Grp78 (Novus Biologicals, Littleton, CO, USA), anti-CHOP (Pierce Biotechnology, Waltham, MA, USA), anti-Herp (Santa Cruz Biotechnology, Santa Cruz, CA, USA) or anti-tubulin antibodies (Sigma-Aldrich, St. Louis, MO, USA). Horseradish peroxidase-conjugated goat anti-rabbit and goat anti-mouse immunoglobulin G (Santa Cruz Biotechnology, Santa Cruz, CA, USA) were used as secondary antibodies. The Enhanced Chemiluminescence System (GE Healthcare, Buckinghamshire, UK) was used for detection.

### Immunofluorescence microscopy

Patient-derived fibroblasts were grown on glass coverslips and fixed with 10% formalin for 20 min at room temperature. The fixed cells were blocked with blocking solution (PBS 1x, 0.1% triton, 5% foetal bovine serum) for 30 min and the cells then incubated overnight at 4°C with anti-GPT antibody (Santa Cruz Biotechnology, Dallas, TX, USA) diluted 1:50 in blocking solution plus anti-calnexin antibody (StressMarq, Victoria, British Columbia) diluted 1:200 in blocking solution. After washing three times with PBS, anti-goat Alexa 555 (Life Technologies, Carlsbad, California) and anti-rabbit Alexa 488 antibodies (Life Technologies, Carlsbad, California) diluted 1:500 in blocking solution were applied for 1 h at room temperature. The cells were then washed with PBS and incubated with DAPI (Merck, Whitehouse Station, NJ, USA) diluted 1:2500 in PBS for 5 min. Samples were observed using an Axiovert200 inverted microscope; photographs were taken using a C9100-02 digital camera (Hamamatsu, Hamamatsu City, Japan).

### Expression of wild type and *DPAGT1* mutants

COS-7 cells were cotransfected with the pCMV6-AC-GFP expression plasmid encoding human *DPAGT1* cDNA (NM_ 001382.3) fused to GFP (RG201787, OriGene, Rockville, MD, USA), and with the Living Color^®^ vector encoding calreticulin fused with DsRed (BD Biosciences, San Jose, Ca, USA). JetPET (Polyplus Transfection, New York, NY, USA) was used as the transfection reagent. Cells were incubated for 48 h and then observed using an Axiovert200 inverted microscope. Photographs were taken using a C9100-02 digital camera (Hamamatsu, Hamamatsu City, Japan). Shadow and background corrections were made before taking photographs to allow quantification of expression using Fiji software.

### RT-PCR and quantitative RT-PCR

Total RNA was isolated using Tripure Isolation Reagent (Life Technologies, Carlsbad, California) following the manufacturer's recommendations. Samples were quantified using a Nanodrop ND-1000 device (Thermo Scientific, Wilmington, MA, USA). cDNA was synthesised by RT-PCR using the High-Capacity cDNA Archive Kit (Life Technologies, Carlsbad, California) in the case of XBP1 RT-PCR and the GeneAmp PCR Amplification System (Applied Biosystems) in the case *ATF4*, *DDIT3* and *HSPA5* qRT-PCR. *XBP1* and *GAPDH* cDNA amplification was performed by PCR using platinum Taq DNA polymerase (Life Technologies, Carlsbad, California) following the manufacturer's instructions, making use of the primers *XBP1* sense (5' TTACGAGAGAAAACTCATGGC 3'), *XBP1* antisense (5' GGGTCCAAGTTG TCCAGAATGC 3'), *GADPH* sense (5' GTCGGAGTCAACGGATTTGG 3') and *GAPDH* antisense (5' TGAGCCCCAGCCTTCTCC 3'). mRNA expression levels of *ATF4*, *DDIT3* and *HSPA5* were determined in control and patient-derived fibroblasts before and after tunicamycin treatment (2.5 μg/ml for 16 h) by qRT-PCR using the LightCycler^®^480 SYBR Green I Master Kit (Roche Applied Sciences), Universal ProbeLibrary probe #88 (*ATF4*), probe #64 (*HSPA5*), and probe #9 (*DDIT3*) (Roche Diagnostics), and employing a LightCycler^®^480 instrument. Data were analysed using Lightcycler^®^ software (Roche Applied Sciences), correlating the initial template concentration with the cycle threshold (Ct) to obtain the relative quantity (RQ) of RNA. The RQ is defined as RQ = 2^−ΔΔCt^, where ΔΔCt is the ΔCt of the patient cell line minus the ΔCt of the control cell line, and ΔCt is the Ct of the target gene minus the Ct of the housekeeping gene (*GAPDH*).

### Statistical analysis

Statistical analyses were performed using IBM SPSS Statistics v.21 software for Windows. The 2-tailed Student t test was used to compare the amount of GPT mutant proteins localised in the ER and the relative mRNA expression level of the UPR genes. Data are reported as means ± SD.

## Results

### Genetic analysis

**Fig 1:**
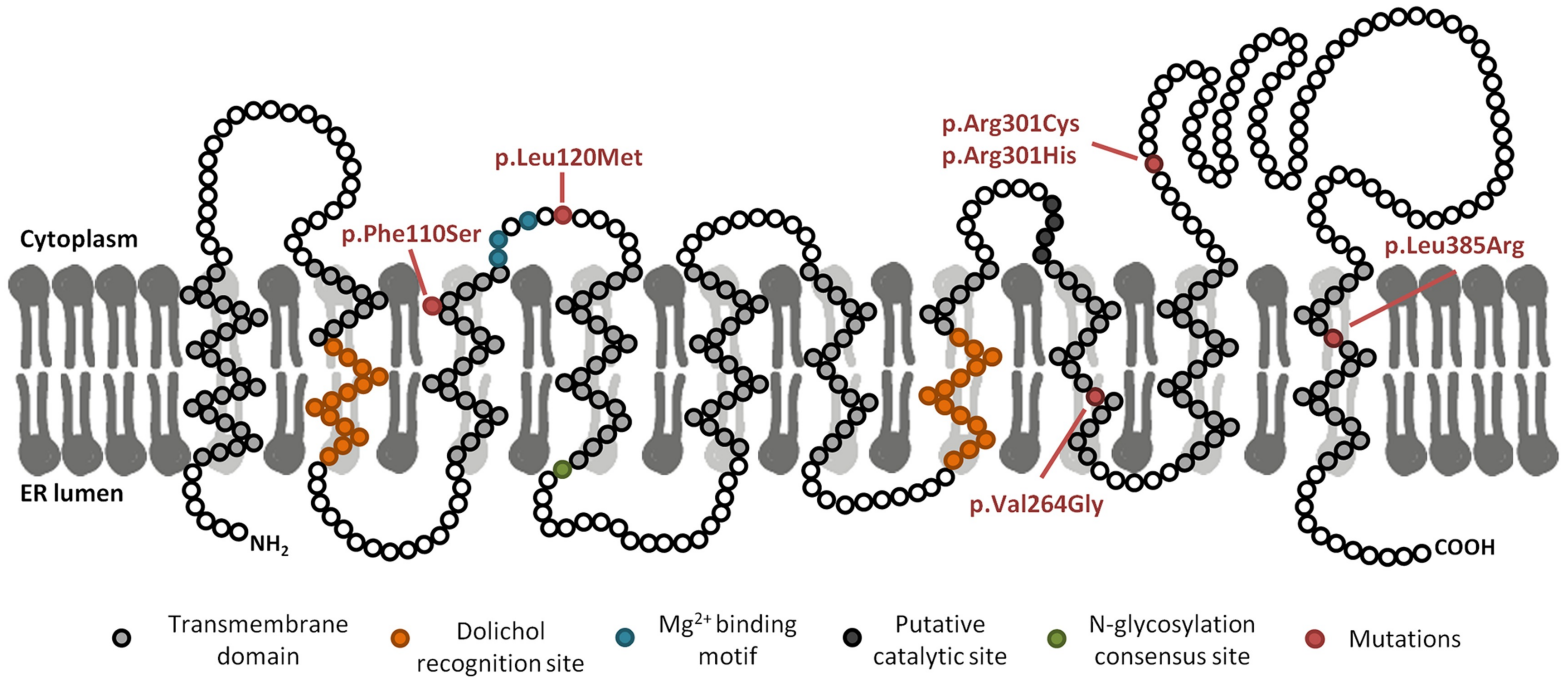
Diagram of GPT protein and location of the reported mutations. Predicted structural model based on the data obtained from The Universal Protein Resource (UniProt), primary accession number Q9H3H5 [17]. Disease-causing mutations included in this work are shown in red.

The following nucleotide changes were detected in *DPAGT1*: c.329T>C (p.Phe110Ser) (described for the first time in this work), c.358C>A (p.Leu120Met), c.791T>G (p.Val264Gly), c.901C>T (p.Arg301Cys), c.902G>A (p.Arg301His) and c.1154T>G (p.Leu385Arg). All three patients were compound heterozygous (Table 1). Fig 1 shows p.Phe110Ser, p.Val264Gly and p.Leu385Arg to be located in intermembrane domains, while p.Le120Met, p.Arg301Cys and p.Arg301His are located in the cytosolic domain of the protein.

Analysis of the predicted effect of these exonic SNVs indicated all to be likely disease-causing mutations since they affected highly or moderately conserved residues. Additionally, all but c.901C>T were predicted to have an effect on the splicing process. The bioinformatic analysis revealed a greater chance of exon skipping for the c.358C>A and c.791T>G mutations due to their potential alteration of an exonic splicing enhancer sequence (Table 2).

**Table 2:** Predicted and observed effect of mutations detected in *DPAGT1* gene.

Mutation		Predicted effect on splicing		Observed effect on splicing	Predicted effect on protein					Observed effect on protein expressed	Likely disease-causing effect
cDNA	Protein	ESEFinder	EX-SKIP		phyloP/alamut	Grantham distance	SIFT	PolyPhen2	Mutation Taster		
c.329T>C	p.Phe110Ser	Potential alteration of splicing. Alteration of an ESE site.	Less chance of exon skipping	Presence of full length transcript	Highly conserved nt and aa	Large	Deleterious	Possibly damaging	Disease causing	Protein localized in the ER 30% reduced	Protein mislocalization
c.358C>A	p.Leu120Met	Potential alteration of splicing. Alteration of an ESE site.	Higher chance of exon skipping	Presence of full length transcript. Recovery of aberrant transcript lacking exon 2 and 3 after cycloheximide treatment	Moderately conserved nt and highly conserved aa	Small	Deleterious	Probably damaging	Disease causing	Protein amount and localization comparable to WT	Splicing alteration and effect on catalytic activity
c.791T>G	p.Val264Gly	Potential alteration of splicing. Creation and alteration of an ESE site.	Higher chance of exon skipping	Lack of full length transcript. Recovery of aberrant transcript lacking exon 7 and 8 after cycloheximide treatment	Highly conserved nt and aa	Moderate	Deleterious	Probably damaging	Disease causing	Protein localized in the ER 40% reduced	Splicing alteration and misfolding
c.901C>T	p.Arg301Cys	No significant splicing motif alteration detected	Comparable chance of exon skipping.	Presence of full length transcript	Moderately conserved nt and highly conserved aa	Large	Deleterious	Probably damaging	Disease causing	Protein amount and localization comparable to WT	Misfolding
c.902G>A	p.Arg301His	Potential alteration of splicing. Creation of an ESE site.	Less chance of exon skipping	Presence of full length transcript	Highly conserved nt and aa	Small	Deleterious	Probably damaging	Disease causing	Protein amount and localization comparable to WT	Misfolding
c.1154T>G	p.Leu385Arg	Potential alteration of splicing. Alteration of an ESE site.	Less chance of exon skipping.	Presence of full length transcript	Highly conserved nt and moderately conserved aa	Moderate	Tolerated	Possibly damaging	Disease causing	Almost completely lack of protein	Misfolding

Predicted data obtained from Alamut visual software (2.7.1 April 2015)

nt: nucleotide; aa: amino acid

ESE: exonic splicing enhancer

**Fig 2:**
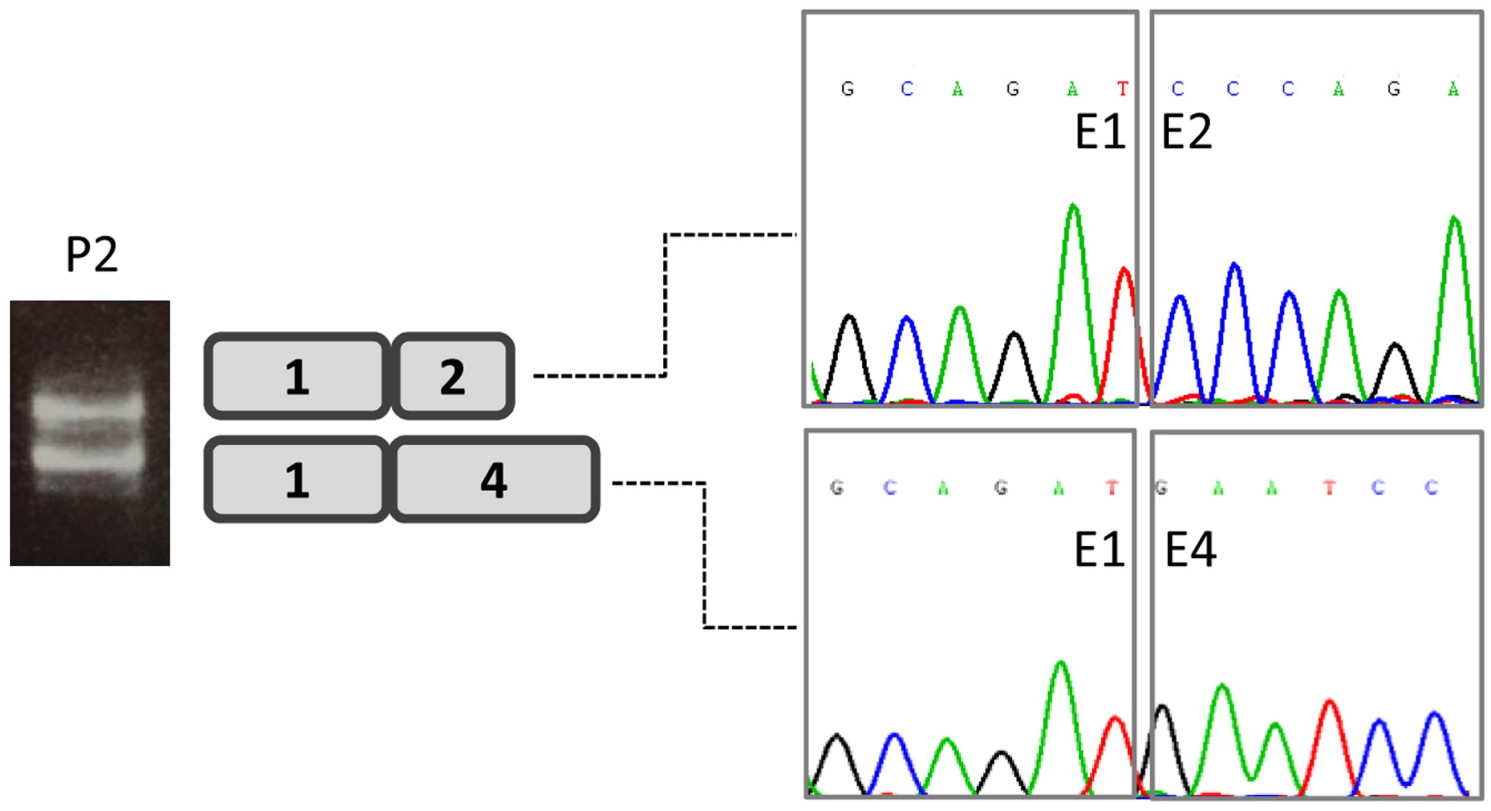
DPAGT1-transcriptional profile analysis of patient 2-derived fibroblasts. Cycloheximide treatment and further sequencing analysis revealed two transcripts, one lacking exons 2 and 3.

*DPAGT1* transcriptional profile analysis of patient-derived fibroblasts revealed the presence of full-length transcripts in all three patients. However, sequence analysis indicated one patient's (patient 2) transcript to carry the c.358C>A mutation only, with no trace of the c.791T>G mutation. Cycloheximide treatment of this patient's fibroblasts allowed the detection of two transcripts, one lacking exons 2 and 3 (probably caused by the effect of the nucleotide change c.358C>A (Fig 2).

**Fig 3:**
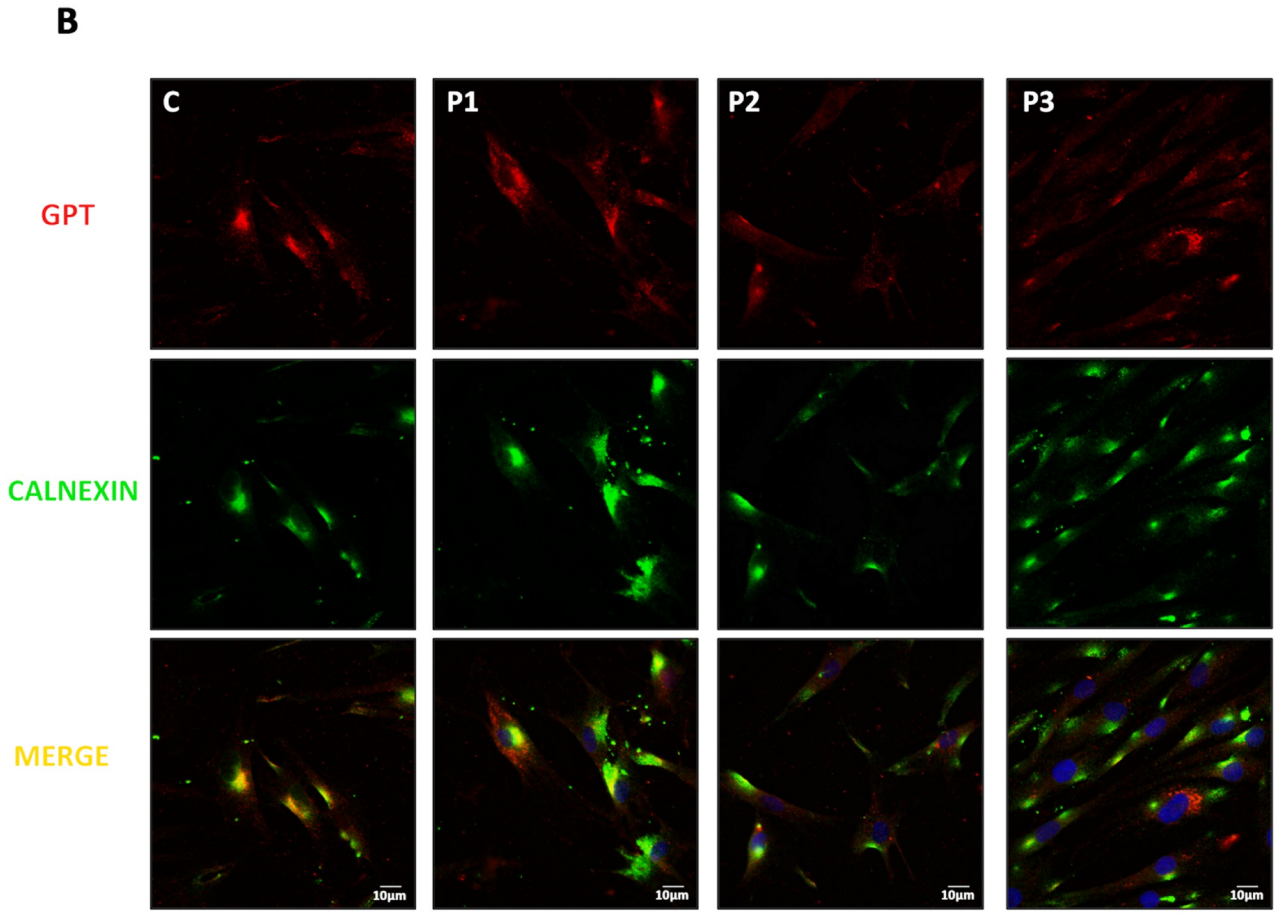
GPT protein level and location in control and DPAGT1-CDG patient-derived fibroblasts. Double labelled immunofluorescence analysis of control (C) and patient cell lines (patients 1, 2 and 3). Cells were double-labelled with GPT (red fluorescence) and Calnexin (green fluorescence) antibodies (ER marker).

Total GPT was determined in all three patients' cells by Western blotting (data not shown). In patients 1 and 3, immunofluorescence (labelling the GPT protein and the ER marker Calnexin) suggested the GPT to be improperly localised in the ER (Fig 3A).

### Functional analysis of the mutations

**Fig 4:**
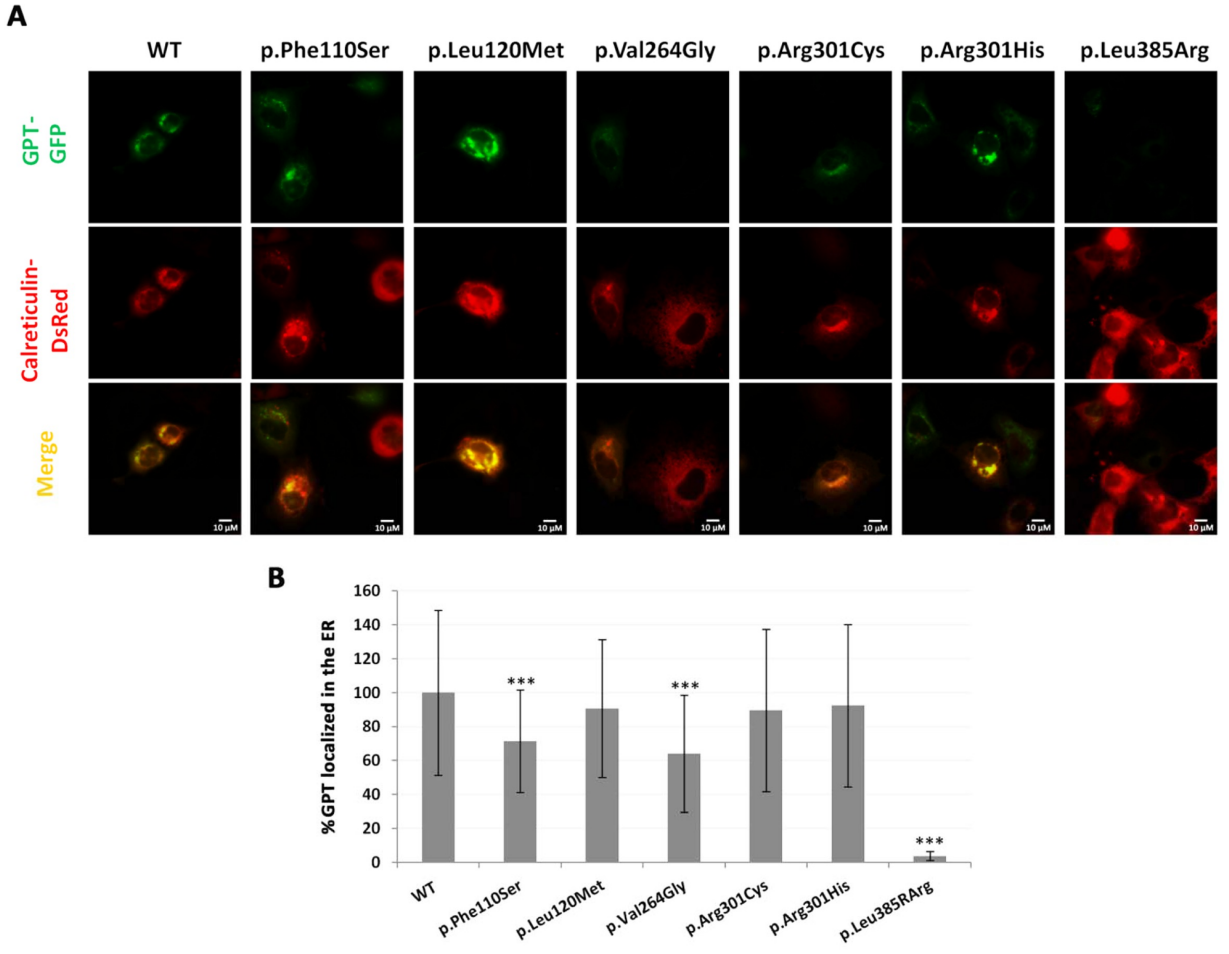
Expression analysis of GTP mutations. A) COS-7 cells were cotransfected with the wild type GPT-GFP fused protein (green fluorescence) or the protein bearing the mutations (p.Phe110Ser, p.Leu120Met, p.Val264Gly, p.Arg301Cys, p.Arg301His and p.Leu385Arg) and with the Calreticulin-DsRed fused protein (red fluorescence) as an ER marker. B) Quantification of GPT-GFP (colocalised with Calnexin-DsRed protein) fluorescence intensity. Data were collected from two different experiments; at least 80 images were analysed. Data represent mean ± SD. ***p<0.001.

To determine the effect of each mutation on GTP stability and the location of mutant proteins in the ER, COS-7 cells were cotransfected with two expression plasmids, one coding for the GFP-GPT fusion protein, the other for calreticulin-DsRed as an ER marker. The effect of c.791T>G (p.Val264Gly) on GTP stability and location was also examined, despite the apparent lack of the full length transcript carrying the mutation, in case some trace of the protein was produced. The results showed the p.Leu385Arg mutant protein to be almost completely absent (Fig 3) suggesting its stability to be severely affected. The p.Phe110Ser and p.Val264Gly mutant proteins were reduced in quantity by 30 and 40% respectively compared to the wild type protein, suggesting them to be less stable or to be incorrectly localised (Fig 3). In contrast, the p.Leu120Met, p.Arg301Cys and p.Arg301His mutant proteins seemed to be stable and properly localised in the ER (Fig 4).

### ER stress analysis

To gain insight into the pathophysiology of DPAGT1-CDG, and the possibility of targeting the UPR as a therapeutic strategy, the activation of genes involved in this response were analysed. Fibroblasts from patients with PMM2-CDG and DPM1-CDG, previously described as showing moderate, chronic UPR activation [8,10,13], were used as positive controls.

**Fig 5:**
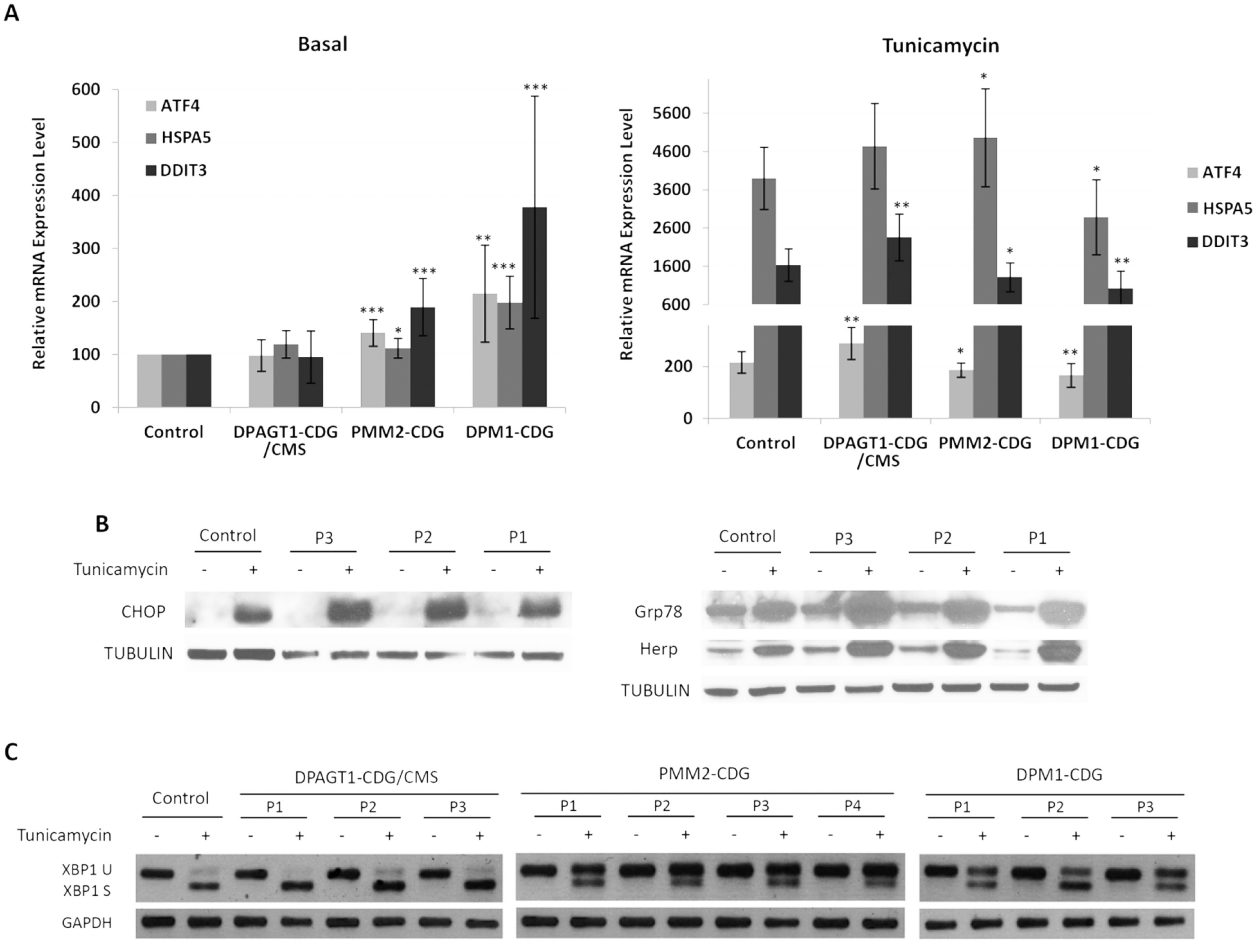
UPR activation in DPAGT1-CDG/CMS, PMM2-CDG and DPM1-CDG patient-derived fibroblasts. A) Quantification of *ATF4*, *HSPA5* (Grp78 protein) and *DDIT3* (CHOP protein) gene expression before and after treatment with tunicamycin (measured by qRT-PCR). Data represent mean ± SD of two controls, three DPAGT1-CDG/CMS, four PMM2-CDG and three DPM1-CDG cell lines. The results are represented as relative mRNA expression compared to the control-derived fibroblasts before treatment. Data were obtained via at least three experiments. *p<0.05; **p<0.01; ***p<0.001. B) Western blot analysis of DPAGT1-CDG patient-derived fibroblasts before and after treatment with tunicamycin. Equal amounts of total soluble protein were loaded onto the SDS-PAGE gel and the proteins immunodetected using anti-CHOP, anti-Grp78, anti-Herp antibodies, with anti-tubulin as a loading control. C) RT-PCR analysis of XBP1 splicing transcription factor in patient-derived fibroblasts untreated and treated with tunicamycin. XBP1 U: Unspliced XBP1 form; XBP1 S: spliced XBP1 form. GAPDH was used as internal control gene ().

The activation of the three UPR signal transducers—the PERK, ATF6 and IRE1 arms- was examined by the study of the relative mRNA expression of *ATF4* (which codes for the protein ATF4), *HSPA5* (which codes for the protein Grp78) and the analysis of XBP1 splicing, respectively. The activation of apoptosis was examined via the relative expression of mRNA levels of *DDIT3* (which codes for the protein CHOP). The results showed no induction of these genes in the DPAGT1-CDG patient-derived fibroblasts (Fig 5A), while the PMM2-CDG- and DPM1-CDG patient-derived cells showed increases in the expression of *ATF4* and *HSPA5* compared to the control cell line. This indicates a moderate activation of the PERK and ATF6 arms of the UPR respectively. Apoptosis in these latter cells was also increased due to an elevated expression of *DDIT3*.

The sensitivity of the three types of patient-derived cells to stressors was analysed via their treatment with tunicamycin, a potent inducer of the UPR. This compound inhibits activity of the GPT protein [14], and thus glycosylation. Compared to control cells, the DPAGT1-CDG patient-derived fibroblasts were found to be more sensitive to tunicamycin, with activation of all three UPR arms. In contrast, the PMM2-CDG and DPM1-CDG patient-derived cells appeared slightly less sensitive to tunicamycin than the control cells (Fig 5A).

The levels of CHOP, Grp78 and Herp (which is also involved in the activation of the UPR) in DPAGT1-CDG patient-derived fibroblasts were analysed before and after tunicamycin treatment. The results confirmed these cells to show no basal UPR activation but greater sensitivity to tunicamycin compared to controls (Fig 5B).

The results for IRE1 arm activation showed the appearance of the spliced *XBP1* form in control and patient-derived fibroblast treated with tunicamycin. However, neither the DPAGT1-CDG, nor PMM2-CDG, or DPM1-CDG patient-derived cells produced this form in the absence of tunicamycin, indicating the IRE arm not to be activated (Fig 5C). Finally, in the DPM1-CDG and PMM2-CDG patient-derived fibroblast, the response to tunicamycin seemed to be weaker than in the control cells.

## Discussion

The comprehensive functional characterization of disease-causing mutations allows tailored therapeutic strategies to be designed. The present work contributes towards the search for therapeutic targets for the treatment of DPAGT1-CDG.

Immunofluorescence analysis revealed GTP to be incorrectly localised in the ER in DPAGT1-CDG patient-derived fibroblasts from two patients (2 and 3). Since the present patients were compound heterozygous, the specific effect of each change had to be elucidated. Mutant expression analysis in the COS-7 expression system, in combination with *DPAGT1* transcriptional profile studies in patient-derived fibroblasts, showed all the studied variants to be pathogenic, either affecting the splicing process, the correct folding of the protein, and/or its subcellular location. Transient expression studies with p.Phe110Ser, p.Val264Gly and p.Leu385Arg cDNA showed the ER to contain reduced amounts of mutant GPT. It is noteworthy that these three residues (Phe110, Val264 and Leu385) are located in the transmembrane domains of the GTP protein, and that the amino acid changes are large, moderate and moderate respectively, as described by the Grantham distance [15]. This indicates a likely effect on the hydrophobicity and/or structure of the corresponding domains, suggesting difficulties may arise in the protein being embedded in the ER membrane. The results for the p.Leu385Arg mutation suggest it to have a strong effect on protein stability, and that it is likely degraded rapidly by the ER-associated protein degradation system. Finally, the mutation p.Phe110Ser seems to affect the correct localization of GPT in the ER.

The *DPAGT1* transcriptional profiles and bioinformatic predictions suggest that the nucleotide change c.358C>A (p.Leu120Met) affects the splicing process since it is located in an exonic splicing enhancer. In agreement, an aberrant transcript lacking exon 2 and 3 was detected. This effect has also been described for the mutation c.360G>C located two nucleotides downstream [16]. The present results showed this mutation to have no effect on GPT stability protein or its localisation in ER. However, it should be noted that the present expression analysis was performed using cDNA, which can reveal no effect on splicing. Indeed, even if the protein was produced, residue Leu120 lies close to the Mg^2+^ binding domain (Fig 1), which has an important role in protein activity. Previous reports describe other disease-causing mutations to lie near this domain [16,17,18,19,20], suggesting that mutation p.Leu120Met likely affects GPT activity.

p.Arg301Cys and p.Arg301His—changes predicted to be harmful by several algorithms [21,22]—were not found in control samples. The results show that they had no effect on splicing, and their transient expressions indicated them to have no effect on protein stability or localization in the ER either. These results conflict, however, with the observed mislocalization of the protein in the fibroblasts of patients 1 and 3. Given the reduced presence of GPT bearing the changes p.Phe110Ser and p.Leu385Arg due to they affect potentially the splicing process or the stability of the protein respectively, the majority of the GPT in these patients’ cells must be of the p.Arg301Cys and p.Arg301His types. The overexpression of p.Phe110Ser and p.Leu385Arg mutant proteins, however, might allow their harmful effect to be overcome [12]. Further work is needed to determine whether these changes really have harmful effects, and the underlying mechanisms that might be involved.

Functional analysis provides insight into the pathogenesis of diseases and can help uncover therapeutic options. With respect to the present destabilizing changes, mutation-specific folding therapies aimed at rescuing protein folding and trafficking might be possible via the use of pharmacological chaperones (PCs) and proteostasis regulators (PRs), which have already shown promise in the treatment of conformational diseases [23]. Research is already underway on a number of PCs and PRs that might rescue cystic fibrosis transmembrane conductance regulator (CFTR), changes which lead to cystic fibrosis [24,25,26]. Indeed, one such PC, Lumacaftor, has been approved by the US Food and Drug Administration for use in combination with the "potentiator" Ivacaftor for the rescue of CFTR channel function (http://pi.vrtx.com/files/uspi_lumacaftor_ivacaftor.pdf).

As have been reported previously, we have detected ER stress in PMM2 and DPM1 patient-derived fibroblasts, but unexpectedly it was not found in the present DPAGT1-CDG patient-derived fibroblasts. In addition to the misfolded hypoglycosylated proteins unable to leave the ER, the accumulation of free and truncated LLO might contribute towards ER stress [11]. LLO analysis of DPM1-CDG cells by other authors [27] showed an accumulation of the truncated LLO form. In PMM2-CDG patient-derived fibroblasts, the ER stress observed might be caused by a subphysiological glucose concentration that induces the accumulation of LLO intermediates [28]. Further, mannose-6-P accumulation in a zebrafish model of PMM2-CDG appears to promote LLO hydrolysis, releasing Dol-P-P, which can then be recycled to make new LLO and free glycans [29]. These glycans accumulate, and might be partly responsible for the observed ER stress. In contrast, no ER stress was observed in the DPAGT1-CDG patient-derived fibroblasts, which might be due to their LLO having a normal structure [17,21]. The present results also showed that the PMM2-CDG and DPM1-CDG patient-derived fibroblasts were less efficient than controls in responding to the ER stress induced by tunicamycin, supporting the idea of ER stress adaptation [10]. However, the DPAGT1-CDG patient-derived fibroblasts seemed to have a more effective response to tunicamycin than the control cells, perhaps because GPT is this stressor's target [14]. Further experiments are needed to determine why these cells are more sensitive to tunicamycin.

In conclusion, this work increases the number of mutations known to be associated with DPAGT1-CDG, and provides bases for developing tailored therapies.

## Supporting information

S1 Table Genotype of PMM2 and DPM1 CDG patient-derived fibroblasts included in the UPR analysis. (DOCX)
