# Supplementary material for: DPAGT1-CDG: Functional analysis of disease-causing pathogenic mutations and role of endoplasmic reticulum stress
Source: PLoS One. 2017 Jun 29;12(6):e0179456. doi: 10.1371/journal.pone.0179456 (PMC5491010; doi:10.1371/journal.pone.0179456)
Supplement: S1 Table — (DOCX) [file pone.0179456.s001.docx]

S1 Table. Genotype of PMM2 and DPM1 CDG patient-derived fibroblasts included in the UPR analysis.

|  | Patient | Genotype |
| --- | --- | --- |
| PMM2-CDG | P1 | c.640-9T>G  c.710C>T (p.Thr237Met) |
|  | P2 | c.640-9T>G  c.193G>T (p.Asp65Tyr) |
|  | P3 | c.97C>T (p.Gln33Ter)  c.131T>C (p.Val44Ala) |
|  | P4 | c.523+3A>G (p.?)  c.484C>T (p.Arg162Trp) |
| DPM1-CDG | P1 | c.742T>C (p.Ser248Pro)  c.742T>C (p.Ser248Pro) |
|  | P2 | c.742T>C (p.Ser248Pro)  c.742T>C (p.Ser248Pro) |
|  | P3 | c.564-1G>A (p.?)  c.742T>C (p.Ser248Pro) |

*PMM2* NM_000303.2; *DPM1* NM_003859.1
